# Early childhood caries in Switzerland: a marker of social inequalities

**DOI:** 10.1186/s12903-015-0066-y

**Published:** 2015-07-22

**Authors:** Stéphanie Baggio, Marcelo Abarca, Patrick Bodenmann, Mario Gehri, Carlos Madrid

**Affiliations:** Life Course and Inequality Research Centre, University of Lausanne, Lausanne, Switzerland; Private practice, University of Lausanne, Lausanne, Switzerland; Vulnerable Population Unit, Department of Ambulatory Care and Community Medicine, University of Lausanne & Lausanne University Hospital, Lausanne, Switzerland; Children’s Hospital, Lausanne University Hospital, Lausanne, Switzerland; Stomatology and Dentistry Unit, Lausanne University Hospital, Lausanne, Switzerland

**Keywords:** Dental health, Early childhood caries, Social inequalities, Socioeconomic background

## Abstract

**Background:**

Early childhood caries (ECC) is a marker of social inequalities worldwide because disadvantaged children are more likely to develop caries than their peers. This study aimed to define the ECC prevalence among children living in French-speaking Switzerland, where data on this topic were scarce, and to assess whether ECC was an early marker of social inequalities in this country.

**Methods:**

The study took place between 2010 and 2012 in the primary care facility of Lausanne Children’s Hospital. We clinically screened 856 children from 36 to 71 months old for ECC, and their caregivers (parents or legal guardians) filled in a questionnaire including items on socioeconomic background (education, occupation, income, literacy and immigration status), dental care and dietary habits. Prevalence rates, prevalence ratios and logistic regressions were calculated.

**Results:**

The overall ECC prevalence was 24.8 %. ECC was less frequent among children from higher socioeconomic backgrounds than children from lower ones (prevalence ratios ≤ 0.58).

**Conclusions:**

This study reported a worrying prevalence rate of ECC among children from 36 to 71 months old, living in French-speaking Switzerland. ECC appears to be a good marker of social inequalities as disadvantaged children, whether from Swiss or immigrant backgrounds, were more likely to have caries than their less disadvantaged peers. Specific preventive interventions regarding ECC are needed for all disadvantaged children, whether immigrants or Swiss.

## Background

Early childhood caries (ECC) is a major health concern worldwide [1, 2]. ECC has been defined as the presence of one or more decayed teeth (with non-cavitated or cavitated lesions), missing teeth (due to caries) or a filled surface on any primary tooth in a child aged 71 months old or younger [3]. ECC is correlated with a high burden of disease [4]: previous studies have associated ECC with deleterious effects on other health outcomes (e.g. poor dental health, but also pain, infection, altered eating habits or sleep disturbances), childhood development (e.g., altered cognitive development, reduced speech development or reduced growth involving low body weight and height) and psychological outcomes for both children and their families (e.g., altered wellbeing and quality of life, poor self-esteem or altered concentration) [5–8].

Despite being largely preventable with good oral health behaviours (e.g., regular dental visits and tooth brushing and using fluoridated toothpaste) and nutritional habits (e.g., low sugar intake, limited night-time bottle-feeding, no sharing of eating utensils and using fluoridated salt or water), ECC remains one of the most common childhood diseases [6, 9–11]. ECC can be understood as “an indicator of preventive missed opportunities” [12].

### A marker of social inequalities

Social inequality is characterized by the existence of unequal opportunities, access or distribution of goods between different social groups within a society [13]. ECC can be seen as a marker of social inequalities because, wherever they live in the world, disadvantaged children are more likely to develop caries than their local peers. Indeed, several studies have highlighted social differences in the prevalence rates of ECC; disadvantaged children also have poorer dental health [14–19]. This disadvantaged population includes children from lower socioeconomic backgrounds, with parents who have lower levels of education and from single-parent families. However, this population also includes children facing a cultural disparity, such as their immigrant status or a language barrier [2, 8, 15–17, 20, 21].

### ECC in Switzerland

ECC prevalence and their associations with socioeconomic factors have been well studied worldwide, yet data are scarce in Switzerland. Overall, dental health is described as improving in Switzerland [22, 23], however, dental care is not covered by the basic health insurance scheme, and patients generally have to pay for their dental treatment. Two recent Swiss studies highlighted that dental care was by far the most common healthcare treatment to be neglected by low-income people facing economic constraints [24, 25]. ECC might, therefore, be expected to be an important marker of social inequalities in Switzerland, and some national studies have already highlighted the relationship between socioeconomic status and dental health [23].

Despite these interesting contextual factors, only one previous study has reported on ECC prevalence rates in Switzerland, and this was in the German-speaking part of the country. A study in Zurich, in 2003, reported that 25.3 % of two- and three-year-old children presented with ECC [26]. This prevalence rate was higher than those reported in other European countries (e.g., Norway, 11 % [18]; Great Britain, 6.8 %–12 % [27]; Greece, 16.5 % [28]; Germany, 7.3 %–20.3 % [29]; Italy, 19 % [12]; France: 18 % [30]). Only Belgium and Spain reported similar ECC prevalence, of 24 % [31] and 28 % [32], respectively. However, data were collected from older children (five-year-olds in Belgium and three- to six-year-olds in Spain). Overall, except for Norway’s, these prevalence rates were higher than the 11 % goal outlined in the US government’s Healthy People 2010 agenda [33].

Thus, the present study’s aims were twofold: 1) to define ECC prevalence in French-speaking Switzerland, where data on this topic were particularly scarce; and 2) to investigate whether ECC might be a strong marker of social inequalities in the country.

## Methods

### Participants

The study was conducted between February 2010 and October 2012 in the primary care facility of Lausanne Children’s Hospital (HEL), a part of the Lausanne University Hospital. The HEL is dedicated entirely to children’s health and is the paediatric reference centre for Lausanne and its suburbs (more than 250,000 inhabitants). It treats all common diseases whether medical, surgical, psychiatric or psychosocial; life-threatening emergencies and pathologies requiring complex technical support are admitted to the main Lausanne University Hospital facility. The HEL’s priorities also include assisting and supporting patients’ families; the institution has a strong social vocation and is rooted in the community. There were more than 53,000 paediatric consultations at the HEL in 2010 (capacity = 29 beds). About 70 % of these patients were immigrants, and the HEL is also involved in the medical monitoring of children without residency permits.

Children were eligible for inclusion in the study if they were between 36 and 71 months old and if their caregiver had a minimum capacity to understand the study’s instructions. Some were admitted to the hospital for regular and emergency medical treatments, and others were there for ambulatory care. However, they all presented minor health problems (no severe or chronically ill children participated). To avoid bias, children coming for dental treatment were not included. A majority of the participants lived near Lausanne.

Following the caregiver’s written informed consent, two dentists clinically screened 856 children for ECC. During a three-month pre-testing period, the two dentists individually screened a number of children for ECC, and their dental assessments were compared. Their scoring was calibrated, and at the end of the pre-test period their convergence rate was 0.99. Examinations took place in the HEL’s primary care waiting room, and although it was impossible to dry their teeth or remove debris in this environment, the children’s teeth were cleaned with sterile gauze. The visual clinical examination of the mouth was performed with the aid of a mobile light. Only deciduous teeth were examined, and the data collected were entered into a specially designed table. Caregivers were also interviewed using a standardised questionnaire available in French, English, Spanish, Albanian and Arabic—the most common languages spoken by those accompanying responsible adults. The dentists also noted any relevant dental or oral pathologies and informed caregivers of them if necessary.

The University of Lausanne Faculty of Biology and Medicine’s Ethics Committee approved the study protocol.

### Sample size

The sample size was calculated in order to detect an odd-ratio of two between two groups—as we had no information about the prevalence rates of socioeconomic variables, the participants were separated at the median into groups with either a lower or a higher socioeconomic background. The non-exposed group (i.e., high socioeconomic background) had a prevalence rate of 10 % ECC, chosen based on the 11 % goal outlined in the US government’s Healthy People 2010 agenda [33]. For a significance level of 0.05, a power of 0.90 and a correction for continuity, each group had to include 403 participants. The minimum total sample size required to assess the associations proposed was thus estimated to 806 participants.

### Measures

*ECC*. A clinical examination of the oral cavity, without the use of X-rays, was performed to establish a dental assessment. The presence of ECC was defined as per the recommendations by Drury et al. [3], and coded dichotomously (‘1’ = presence, ‘0’ = absence). Due to the examination conditions, only frank cavitation was coded 1.

#### Socioeconomic background

Socioeconomic background included individual-level measures:

1. Parents’ level of education: incomplete compulsory education, compulsory education, apprenticeship (vocational school), secondary education (high school diploma), tertiary education (university) (for similar cut-off points, see examples in [34, 35]). The highest level of education of the two parents was used.
2. Parents’ professional level: we selected three classes from the UK’s National Statistics Socioeconomic Classifications [36], i.e., higher occupations (e.g., senior manager), intermediate occupations (e.g., manager), lower occupations (e.g., employee, manual worker), and added classes that did not fit into those above, i.e., self-employed and unemployed. The highest professional level of the two parents was used.
3. Family income: low family income (less than CHF 4,000, i.e., around USD 4,270), medium family income (between CHF 4,000 and CHF 6,000, i.e., around USD 6,400), high family income (more than CHF 6,000), or did not want to answer (for similar cut-off points, see examples in [37]).
4. Parents’ French literacy: this was coded ‘1’ for a good understanding and ‘0’ otherwise.
5. The 2011 United Nations human development index (HDI) was used to assess immigration status and categorize families into two groups, i.e., immigrants from countries with HDI ≥ 0.8 (developed countries) and people from Switzerland, and immigrants from countries with HDI < 0.8 (developing countries) [38]. Immigration status was coded ‘1’ if at least one parent came from a developed country and ‘0’ otherwise. A subgroup was also created for children whose parents were both Swiss citizens.

#### Covariates

Children’s age and gender were recorded. Knowledge and attitudes related to dental care were assessed using: 1) the most recent visit to a dentist (coded ‘1’ for at least one check-up during the previous 12 months, ‘0’ otherwise); 2) the child’s average frequency of tooth brushing (coded ‘1’ for brushing three times per day, ‘0’ otherwise); 3) the caregiver’s average frequency of tooth brushing (coded ‘1’ for brushing three times per day, ‘0’ otherwise); 4) the caregiver’s presence during the child’s most recent tooth brushing (coded ‘1’ if a caregiver was present, ‘0’ otherwise); 5) parents in agreement about child’s tooth brushing practices (coded ‘1’ if parents supported each other, ‘0’ otherwise); 6) how the child fell asleep the previous night (with water or nothing, with milk, or with a sugar-based drink); and 7) information given by the paediatrician regarding ECC (coded ‘1’ when parents had received information, ‘0’ otherwise).

### Statistical analyses

#### ECC prevalence in Switzerland

The prevalence rate of ECC and descriptive statistics for covariates were calculated. Bivariate associations between ECC and covariates were tested using exact Fischer tests.

ECC prevalence and bivariate associations for the subsample of children whose parents were both Swiss citizens (excluding immigrants) were additionally calculated to test whether the relationship between ECC and socioeconomic background was still the same, and to test whether the result was not due only to immigration status. Analyses included parents’ level of education, parents’ professional level, and family income, because literacy problems and immigration were absent within this subsample. Incompleted compulsory education and unemployed were excluded from the analysis because of their very low sample size.

Prevalence ratios and 95 % confidence intervals were computed for socioeconomic variables, using the lowest level (i.e., incomplete compulsory education, unemployed, low family income, not good French understanding, and immigrants from countries with HDI ≥ 0.8) as the reference category. A log-binomial model was run to compute prevalence ratios and their significance level.

#### Multivariate associations between ECC and socioeconomic background

Subsequently, multivariate analyses were performed. Logistic regressions were calculated using ECC as a dependent variable and socioeconomic background factors as independent variables. Since the socioeconomic variables shared a large amount of common variance, five different logistic regressions were performed (parents’ level of education, parents’ professional level, family income, parents’ literacy, and parents’ immigration status). Covariates were included as control variables (i.e., age, gender, dental check-ups, child’s frequency of tooth brushing, caregiver’s frequency of tooth brushing, parental presence at most recent tooth brushing, parents in agreement about child’s tooth brushing, how the child fell asleep the previous night, and information given by the paediatrician). For each model, a McFadden pseudo R-squared was calculated to estimate the effect size of each socioeconomic variable of interest. Pairwise comparisons were calculated to compare different levels of the categorical socioeconomic variables. The length of time that migrants had been in the country was controlled for. As the results concerning ECC were the same whether or not this confounder was included, the analyses were carried out without this covariate.

Descriptive statistics and logistic regressions were carried out using Stata version 14 software.

## Results

### ECC prevalence

The children’s average age was 4.42 ± 0.88 years old. The prevalence rate of ECC was 24.8 %. Bivariate associations are summarized in Table 1 (demographics and covariates) and Table 2 (socioeconomic backgrounds). With regard to demographics and covariates, there were significant differences in ECC prevalence rates associated with variables related to parental attitudes and behaviours. Children whose parents frequently brushed their own teeth, who were present at the most recent tooth brushing, and who agreed on their children’s tooth-brushing practices had a lower ECC prevalence. On the contrary, however, information from a paediatrician was not associated with a decrease in ECC prevalence, nor was an annual visit to a dentist.

**Table 1:** Descriptive statistics for demographics and covariates, and bivariate associations with ECC

Demographics and covariates		% (N)	% of ECC^1^
Gender			
	Boys	55.6 (476)	25.6^a^
	Girls	44.4 (380)	23.7^a^
Dental check-up in previous 12 months			
	Yes	44.9 (384)	35.9^a^
	No	55.1 (472)	15.7^b^
Child’s frequency of tooth brushing			
	<3 times a day	74.2 (635)	23.6^a^
	3 times a day	25.2 (216)	27.8^a^
	Missing	0.6 (5)	-
Parental frequency of tooth brushing			
	< 3 times a day	65.2 (558)	29.9^b^
	3 times a day	33.2 (284)	21.7^a^
	Missing	1.6 (14)	-
Parental presence at most recent tooth brushing			
	Yes	86.7 (742)	22.9^a^
	No	12.9 (110)	37.3^b^
	Missing	0.5 (4)	-
Parents in agreement on child’s tooth brushing practices			
	Yes	84.5 (723)	23.1^a^
	No	14.8 (127)	34.6^b^
	Missing	0.7 (6)	-
Way children fell asleep previous night			
	With water/nothing	72.0 (616)	26.0^a^
	With milk	24.5 (210)	21.0^a^
	With sugar-based drink	2.6 (22)	22.7^a^
	Missing	0.9 (8)	-
Information given by pediatrician regarding ECC			
	Yes	48.5 (415)	23.4^a^
	No	51.3 (439)	26.0^a^
	Missing	0.2 (2)	-

^1^Row percentages. For example: A total of 25.6 % of the boys had ECC

^a, b^For significant Fischer’s exact test, a same subscript letter within a column denotes that proportions did not differ; two different subscript letters denote that proportions differed at the 0.05 level

**Table 2:** Descriptive statistics for socioeconomic background and bivariate associations with ECC

Socioeconomic background		% (N)	% of ECC^1^	PR of ECC^2^
Parents’ level of education				
	Incomplete compulsory education	14.6 (14)	64.3^a^	Reference category
	Compulsory education	15.4 (132)	31.1^b^	0.48 [0.30-0.77]^**^
	Apprenticeship (vocational school)	22.4 (192)	28.1^b^	0.44 [0.28-0.69]^***^
	Secondary education (high-school diploma)	18.2 (156)	31.4^b^	0.49 [0.31-0.77]^**^
	Tertiary education (university)	42.1 (360)	15.8^c^	0.25 [0.16-0.39]^***^
	Missing	0.2 (2)	-	-
Parents’ professional level				
	Unemployed	6.8 (58)	48.3^a^	Reference category
	Lower occupations	55.3 (473)	27.9^b^	0.48 [0.25-0.90]^*^
	Intermediate occupations	16.8 (144)	17.4^c^	0.58 [0.43-0.78]^***^
	Higher occupations	16.5 (141)	12.8^c^	0.36 [0.23-0.56]^***^
	Self-employed	4.6 (39)	23.1^b,c^	0.26 [0.16-0.44]^***^
	Missing	0.1 (1)	-	
Family income				
	Low (< CHF 4,000)	18.1 (155)	42.6^a^	Reference category
	Medium (CHF 4,000 to CHF 6,000)	25.1 (214)	20.1^b^	0.52 [0.38-0.71]^***^
	High (> CHF 6,000)	7.6 (65)	13.8^b^	0.33 [0.17-0.61]^***^
	Other	15.5 (133)	18.8^b^	0.44 [0.30-0.66]^***^
	Did not want to answer	33.4 (286)	22.7^b^	0.53 [0.40-0.71]^***^
	Missing	0.4 (3)	-	
Parents’ literacy				
	Problems	20.4 (175)	41.7^a^	Reference category
	No problems	79.6 (681)	20.4^b^	0.49 [0.39-0.62]^***^
Parents’ immigration status				
	Developing countries (HDI < 0.8)	29.7 (254)	42.5^a^	Reference category
	Developed countries (HDI > 0.8)/Swiss citizens	70.2 (601)	17.1^b^	0.40 [0.32-0.51]^***^
	Missing	0.1 (1)	-	-

ECC: Early Childhood Caries; PR: Prevalence Ratio

^1^Row percentages. For example: A total of 14.6 % of the children whose parents had an incomplete compulsory education had ECC

^2^PR were computed using log-binomial models

^a, b, c^For significant Fischer’s exact test, a same subscript letter within a column denotes that proportions did not differ; two different subscript letters denote that proportions differed at the 0.05 level

^*^ *p* < .05, ^**^ *p* < .01, ^***^ *p* < .001

Results showed higher rates of ECC among children from lower socioeconomic backgrounds than those from higher ones, and this for all five variables. For example: with parents who had not completed their compulsory education, ECC = 64.3 %; but with parents with a tertiary education, ECC = 15.8 % (PR = 0.25); with unemployed parents, ECC = 48.3 %; but with parents with a high professional level, ECC = 16.0 % (PR = 0.44); in lower-income families, ECC = 42.6 %; but in higher-income families, ECC = 12.8 % (PR = 0.33); with parents with literacy problems, ECC = 41.7 %; but with no literacy problems: ECC = 20.4 % (PR = 0.49); and with parents from a developing country, ECC = 42.5 %; but with parents from a developed country, ECC = 17.1 % (PR = 0.40).

In addition, we also computed bivariate associations of ECC prevalence with socioeconomic backgrounds of Swiss children with two Swiss parents (n = 457). In this group, the prevalence rate of ECC was 12.1 %, whereas the prevalence rate for immigrant children was 38.6 %. The results showed that even though ECC prevalence was lower, there was still an association between ECC and the socioeconomic background of the subgroup of children with two Swiss parents. Regarding parents’ level of education, children with parents with a tertiary education had a significant lower ECC prevalence (6.1 %, p < .05) in comparison with other levels (compulsory education: 19.6 %, apprenticeship: 16.4 %, secondary education: 21.9 %). Regarding parents’ professional level, children whose parents had a higher occupation had a significantly lower ECC prevalence (13.6 %, p < .05) in comparison with other levels (lower occupations: 20.0 %, intermediate occupations: 14.7 %, but not with self-employed: 6.6 %). Finally, family income was also associated with ECC prevalence: children with a low family income were more likely to have ECC (24.4 %, p < .05) in comparison with other levels (medium: 13.0 %, high: 11.1 %, other: 11.6 %, no answer: 10.1 %).

### Multivariate associations of ECC with socioeconomic background

Table 3 shows the adjusted estimate proportions of ECC according to socioeconomic variables and controlling for covariates. All variables were significantly associated with ECC (p < .001). The parents’ immigration status had the highest effect size (7.74 %), whereas other socioeconomic variables explained between 3.07 % and 3.54 % of the variance in ECC. The complete models, including dental care and dietary habits, explained between 10.70 % and 14.96 % of the variance in ECC.

**Table 3:** Logistic regressions of ECC on socioeconomic variables

Socioeconomic background		Adjusted estimated prevalence	Likelihood ratio test p-value	Effect size for socioeconomic variable of interest^1^	Effect size for the complete model^1^
Parents’ level of education			< .001	3.14 %	10.70 %
	Incomplete compulsory education	0.61^a^			
	Compulsory education	0.25^b^			
	Apprenticeship (vocational school)	0.23^b^			
	Secondary education (high school diploma)	0.25^b^			
	Tertiary education (university)	0.13^c^			
Parents’ professional level			< .001	3.74 %	11.26 %
	Unemployed	0.42^a^			
	Lower occupations	0.23^b^			
	Intermediate occupations	0.12^c^			
	Higher occupations	0.10^c^			
	Self-employed	0.17^b,c^			
Family income			< .001	3.35 %	10.94 %
	Low (< CHF 4,000)	0.36^a^			
	Medium (CHF 4,000 to CHF 6,000)	0.17^b^			
	High (> CHF 6,000)	0.10^b^			
	Other	0.15^b^			
	Did not want to answer	0.18^b^			
Parents’ literacy			< .001	3.45 %	11.00 %
	Problems	0.37^a^			
	No problems	0.17^b^			
Parents’ immigration status			< .001	7.74 %	14.96 %
	Developing countries (HDI < 0.8)	0.40^a^			
	Developed countries (HDI > 0.8) and Swiss citizens	0.13^b^			

^a, b, c^For significant pairwise comparisons, a same subscript letter within a column denotes that proportions did not differ; two different subscript letters denote that proportions differed at the 0.05 level

^1^Effect size measured with the McFadden pseudo R-square. The whole model included all demographics and covariates

As reported in the bivariate analyses, ECC was more likely to occur in children from lower socioeconomic backgrounds, with adjusted estimated proportions ranging from .36 to .61. Conversely, children from higher socioeconomic backgrounds were less likely to have ECC, with adjusted estimated proportions ranging from .10 to .17.

## Discussion

This study aimed to provide the ECC prevalence among children living in French-speaking Switzerland and to assess whether socioeconomic background had a significant influence on the risk of ECC, as it does in other countries. In other words, ECC was tested to ascertain whether it was an early marker of social inequalities.

### ECC prevalence in French-speaking Switzerland

The prevalence rate of ECC in the children examined, from 36 to 71 months old, was unexpectedly high: 24.8 % had at least one decayed primary tooth, a primary tooth missing due to caries, or a filled primary tooth surface. This prevalence rate was higher than those reported in numerous other European countries: from 6.8 % to 19 % [12, 18, 27–30]. ECC was particularly prevalent among young children living in French-speaking Switzerland, even though dental health in the country has been described as improving [23]. One reason may be that Switzerland has a high immigration rate—the highest among European countries [39, 40]. In 2010, the proportion of immigrants in Switzerland was 23 %, far higher than the second highest proportion, in Germany, with 11.9 % of immigrants [41]. Many studies have reported that migrants have higher ECC prevalence, including in Switzerland [26]. Indeed, the prevalence rate of ECC for the subsample of children whose parents were both Swiss was only 12.7 %, although this result was still higher than that reported by Menghini et al. [26], who calculated a prevalence rate of ECC of 7.5 % among Swiss children.

### ECC as a marker of social inequalities and the importance of immigration status

The present study highlighted social inequalities related to dental health. As socioeconomic background is a multi-dimensional phenomenon, including several sources of social differences, we used multi-component indicators [16]. The effects of social differences are often investigated using geographical measures, focusing on disadvantaged areas or deprived communities [42]. However, this approach does not capture the entire extent of inequalities [42], and studies dealing with individual-level measures are needed [16, 31]. The results presented here show that in French-speaking Switzerland, ECC is significantly related to socioeconomic background, with both a higher proportion and prevalence ratio of ECC among disadvantaged children. This result is in line with those reported in previous studies in other countries [2, 8, 15–17, 20, 21]. Disadvantaged children included those whose parents had not completed primary education (61 % presented with ECC after controlling for covariates) or were unemployed (41 %), those from lower-income families (36 %), those whose parents had literacy problems or faced a language barrier (37 %), and those whose parents came from a developing country (40 %).

The most important effect size was related to the parents’ immigration status: this explained twice as much of the percentage of variance of ECC as other socioeconomic variables. Thus, immigration may be a major contributing factor to ECC in Switzerland, a country with a high immigration rate. These results were in accordance with the high ECC prevalence among immigrants in Zurich, in German-speaking Switzerland, (38.5 %) reported by Menghini et al. [26]. Immigrants may have different ideas and beliefs about oral health, infant feeding practises and oral health awareness [43]. Previous epidemiological studies and clinical surveys have suggested links between race/ethnicity and oral health status [44], however, the specific actual cultural beliefs and values that influence decisions or practices regarding oral health are seldom reported. Further research into immigrants’ cultural beliefs and their associations with their behaviours and practices surrounding oral health and seeking dental care would provide information helpful in designing future preventive and treatment programmes. Butani et al. [44] suggested that qualitative research would be suitable for this purpose, providing valuable information towards understanding cultural beliefs related to oral health and helping to explore cultural reasons for seeking or delaying dental care. They also suggested that a community-based participatory approach [45] would help to involve community members, understand cultural beliefs and practises, and implement appropriate interventions.

In order to remove the influence of Switzerland’s particularly significant immigrant population on ECC prevalence, we also tested the association between ECC and the socioeconomic background of children whose parents were both Swiss. Although the prevalence rate of ECC in this subsample was much lower than that reported for the entire cohort, the association with disadvantaged backgrounds remained. Thus, children of Swiss parents with a lower socioeconomic status (lower family income, lower level of education, lower occupations) were more likely to have ECC than their more advantaged peers. ECC remained a marker of social inequalities for Swiss citizens, especially of harsh social inequalities affecting vulnerable populations [12]. The high ECC prevalence, even among disadvantaged Swiss children, may reflect economic difficulties and a resultant renunciation of healthcare [24, 25]. Menghini et al. [26] recommended specific preventive interventions for immigrants because they had the higher prevalence rate of ECC. The results of our study show that disadvantaged Swiss children should also be included in such preventive programs.

### Preventing ECC

ECC is a preventable disease, so early interventions should be made to identify high-risk children and thus avoid it. A simple way to provide families with information about ECC is to help paediatricians and other professionals caring for children to recognize the risks of ECC [46]. Moreover, since good parental practises regarding oral health were associated with lower prevalence rates of ECC, preventive intervention should also target parents (e.g., their own oral health and the importance they give to their children’s oral health).

### Limitations

This study had some limitations. Firstly, its cross-sectional nature did not allow us to draw any causal paths between ECC and factors associated with it. Secondly, the use of a self-administered questionnaire for caregivers may have induced either a response or recall bias. Finally, generalising these results to the whole country should be done with caution since the sample was based on children living in western Switzerland who had consulted a paediatrician in a primary care hospital. Moreover, the patients visiting the HEL included a high proportion of immigrants (70 %), and even though the HEL is the paediatric reference centre for Lausanne and its suburbs, our results may reflect the large number of immigrants who came to the HEL. Europeans make up the majority of immigrants in Lausanne and its suburbs (in 2011, 83 % of immigrants were from Europe, 72 % from the European Union [47]). In 2010, at 30.5 %, the canton of Vaud (where Lausanne is located) had one of the highest immigration rates in the country [48], whereas it was 23 % for Switzerland as a whole [41]. Our results may, therefore, be representative of the particularities of this part of western Switzerland. Indeed, the ECC prevalence was similar to one reported among children living in Zurich, allowing the authors to be confident about the representativeness of the results. Again, with regards to the sample, children consulting for dental healthcare problems were excluded. This could have had an impact on the findings in two ways: children who consulted for ECC problems could have been those whose lack of dental hygiene had let caries go too far (perhaps from families of a lower socioeconomic background), or they could have been children who had regular preventive dental care (perhaps from families of a higher socioeconomic background). Finally, the conditions of the examination (with no drying or cleaning of teeth) may have resulted in an underestimation of ECC, because only frank cavitation was recorded.

## Conclusion

In conclusion, this study reported a worrying ECC prevalence among children aged 36 to 71 months old, living in French-speaking Switzerland, and this despite the country’s undoubted overall wealth and good health. ECC was shown to be a marker of social inequalities in the country, as disadvantaged children, whether from a Swiss or immigrant background, were more likely to have caries than their more advantaged peers. However, a deeper understanding of how health behaviours are influenced by culture, health beliefs, acculturation and attitudes is needed in order to formulate appropriate oral health promotion policies for specific groups of Switzerland’s population.

## Abbreviation

ECC: Early childhood caries
